# Nitrogen, manganese, iron, and carbon resource acquisition are potential functions of the wild rice *Oryza rufipogon* core rhizomicrobiome

**DOI:** 10.1186/s40168-022-01360-6

**Published:** 2022-11-22

**Authors:** Jingjing Chang, Lei Tian, Marcio F.A. Leite, Yu Sun, Shaohua Shi, Shangqi Xu, Jilin Wang, Hongping Chen, Dazhou Chen, Jianfeng Zhang, Chunjie Tian, Eiko E. Kuramae

**Affiliations:** 1 Key Laboratory of Mollisols Agroecology, Northeast Institute of Geography and Agroecology, Chinese Academy of Sciences, Changchun, 130102 Jilin China; 2 University of Chinese Academy of Sciences, Beijing, 100049 China; 3 Department of Microbial Ecology, Netherlands Institute of Ecology NIOO-KNAW, 6708 PB Wageningen, the Netherlands; 4 Ecology and Biodiversity, Institute of Environmental Biology, Utrecht University, 3584 CH Utrecht, the Netherlands; 5 Jiangxi Super-rice Research and Development Center, National Engineering Laboratory for Rice, Nanchang, China; 6 College of Life Science, Jilin Agricultural University, Changchun, Jilin China

**Keywords:** In situ, Ex situ, Nitrogen use efficiency, Free-living N fixers, Dongxiang wild rice, Rhizosphere microbiome

## Abstract

**Background:**

The assembly of the rhizomicrobiome, i.e., the microbiome in the soil adhering to the root, is influenced by soil conditions. Here, we investigated the core rhizomicrobiome of a wild plant species transplanted to an identical soil type with small differences in chemical factors and the impact of these soil chemistry differences on the core microbiome after long-term cultivation. We sampled three natural reserve populations of wild rice (i.e., in situ) and three populations of transplanted in situ wild rice grown ex situ for more than 40 years to determine the core wild rice rhizomicrobiome.

**Results:**

Generalized joint attribute modeling (GJAM) identified a total of 44 amplicon sequence variants (ASVs) composing the core wild rice rhizomicrobiome, including 35 bacterial ASVs belonging to the phyla Actinobacteria, Chloroflexi, Firmicutes, and Nitrospirae and 9 fungal ASVs belonging to the phyla Ascomycota, Basidiomycota, and Rozellomycota. Nine core bacterial ASVs belonging to the genera *Haliangium*, *Anaeromyxobacter*, *Bradyrhizobium*, and *Bacillus* were more abundant in the rhizosphere of ex situ wild rice than in the rhizosphere of in situ wild rice. The main ecological functions of the core microbiome were nitrogen fixation, manganese oxidation, aerobic chemoheterotrophy, chemoheterotrophy, and iron respiration, suggesting roles of the core rhizomicrobiome in improving nutrient resource acquisition for rice growth. The function of the core rhizosphere bacterial community was significantly (*p* < 0.05) shaped by electrical conductivity, total nitrogen, and available phosphorus present in the soil adhering to the roots.

**Conclusion:**

We discovered that nitrogen, manganese, iron, and carbon resource acquisition are potential functions of the core rhizomicrobiome of the wild rice *Oryza rufipogon*. Our findings suggest that further potential utilization of the core rhizomicrobiome should consider the effects of soil properties on the abundances of different genera.

Video Abstract

**Supplementary Information:**

The online version contains supplementary material available at 10.1186/s40168-022-01360-6.

## Background

The rhizosphere comprises the soil adhering to the root up to a distance of 1 mm. The plant directly stimulates microbiome assembly in the rhizosphere [1–4], as confirmed by studies of the impact of land-use changes on rhizosphere microbial assembly in soils ranging from tropical forests in the Amazon to agricultural fields. There is also evidence that plant genetic makeup influences microbiome assembly in the rhizosphere. Oyserman et al. [5] reported that specific tomato quantitative trait loci are associated with bacterial genes involved in the metabolism of plant polysaccharides, iron, sulfur, trehalose, and vitamins, and Deng et al. [6] found that sorghum plant loci control heritability of the rhizosphere microbiome. Furthermore, the rhizosphere microbiome is influenced by soil type, as soil chemical and physical characteristics (e.g., soil pH, texture, and nutrient availability) shape microbiome assembly within the rhizosphere [7–12]. For example, the use of lime alone or in combination with gypsum to decrease soil acidity increases the abundances of genes with specific roles in nitrogen fixation and decreases the abundances of genes involved in nitrification and denitrification in the soil and rhizosphere of grasses (ruzigrass and maize) [13]. In our recent review [14], we proposed that a proper understanding of the dynamics of rhizosphere microbial assembly requires detailed knowledge of the abiotic conditions present in the immediate vicinity of the growing root, such as the pH and nutrient properties of the soil surrounding the root. However, the only study to focus on the effect of soil conditions surrounding the root on rhizosphere microbial (bacterial and fungal) assembly is that by Ceja-Navarro et al. [15], who demonstrated that the diversity and composition of the rhizosphere protist communities of switchgrass plants are influenced by environmental properties such as the pH of the soil adhering to the root.

The rhizosphere microbiome provides several beneficial functions for host plants, such as improving mineral nutrient absorption and enhancing resistance to soil-borne pathogens [16–20]. The recruitment of a core rhizosphere microbiome with beneficial functions may also depend on plant genotype [12, 21]. However, tools for identifying the core microbes assembled in the rhizosphere remain largely unsystematic. A variety of methods have been used to identify and measure the core microbiome, including identifying classified groups of closely related individuals based on amplicon sequence variants (ASVs) or operational taxonomic units (OTUs) that are shared among microbial consortia in all treatments [22, 23]. Recently, Rolando et al. [24] determined the core root and rhizosphere microbiome of *Spartina alterniflora* by analyzing the accumulated richness and relative abundance using species prevalence cutoff thresholds. One drawback of these methods for determining the core microbes that are consistently selected by a specific plant genotype, e.g., Venn diagrams, is that they only identify ASVs or OTUs that are detected in all treatments [22]. In previous work, we circumvented this problem by using joint species distribution modeling [25, 26], namely, generalized joint attribute modeling (GJAM), to determine the microbiome profiles of different treatments. In this current study, we evaluated the same plant genotype under distinct environmental conditions to identify microbes that are consistently assembled in the rhizosphere regardless of environmental conditions, which we denote as the core selection. This approach provides a new opportunity to detect ecologically relevant microbes that form the core microbiome of a rice genotype.

The genome of Dongxiang wild rice (*Oryza rufipogon*), a perennial grass, contains multiple cold resistance loci that allow this species to grow in more northern locations than other rice genotypes [27]. Since the first discovery of primitive populations of Dongxiang wild rice in 1980, researchers have protected the ecogeographical distribution of this species in Jiangxi Province, China, using fences and other in situ measures to prevent interference from humans, cattle, and sheep [28, 29]. Wild rice populations from the primitive populations of Dongxiang wild rice have also been transplanted in different locations, and an ex situ artificial protection nursery has been established at the Jiangxi Academy of Agricultural Science in Nanchang, Jiangxi Province, China. The transplanted Dongxiang wild rice has been maintained ex situ for 40 years as a permanent grass by mowing before the seeding phase. The soil types of the in situ and ex situ wild rice populations are the same, and they are located in the same province, but there are small differences in soil physicochemical conditions.

The impact of plant stage and age on the rhizosphere microbiome has frequently been disregarded. Intriguingly, the impact of the long-term plant growth legacy on shaping the rhizosphere microbiome is greater than the impact of plant age [30]. In this study, we took advantage of the 40-year history of transplantation and maintenance of Dongxiang wild rice ex situ to ask the following questions: (i) Does this rice genotype carry a core microbiome? If so, (ii) are the potential functions of the core microbiome related to plant growth? To answer these questions, we determined the core rhizosphere bacterial and fungal communities by GJAM analysis and inferred the core microbiome’s potential functions using the FAPROTAX database. The bacterial and fungal communities were identified by amplicon sequencing of the partial 16S rRNA gene and the internal transcribed spacer (ITS), respectively.

## Results

### Dongxiang wild rice (*Oryza rufipogon*) rhizomicrobial diversity and core rhizomicrobiome

After quality filtering of the sequences, the average (5 replicates) number of non-chimeric reads of bacteria for each population was 43,600 for AJSI, 81,357 for STSI, 83,122 for ZTI, 49,137 for AJS, 62,926 for STS, and 52,638 for ZT (Table S1). The average (5 replicates) number of non-chimeric reads of fungi for each population was 122,913 for AJSI, 33,164 for STSI, 23,020 for ZTI, 116,417 for AJS, 24,265 for STS, and 143,622 for ZT. The alpha diversity (Chao1 and Shannon indices) of the rhizosphere bacterial and fungal communities of the different populations of ex situ wild rice were significantly higher than those of the in situ populations (Fig. S1, *p* < 0.05). Principal coordinate analysis (PCoA) based on Bray–Curtis dissimilarity showed that both the bacterial and fungal communities in the rhizosphere of the three in situ natural reserve wild rice populations clustered individually, while the bacterial and fungal communities in the rhizosphere of ex situ wild rice each formed a single cluster (Fig. S2, PERMANOVA, bacteria: *R*^2^ = 0.71, *p* < 0.001; fungi: *R*^2^ = 0.81, *p* < 0.001).

**Fig. 1:**
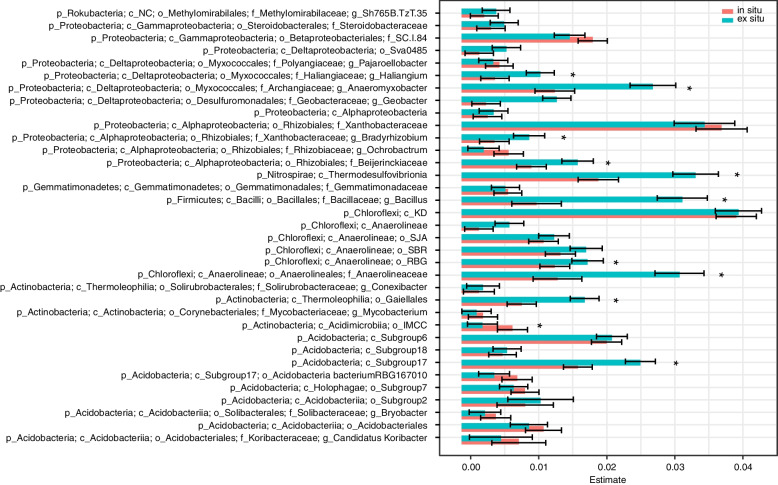
The core rhizosphere bacteria of wild rice grown in situ and ex situ. Generalized joint attribute modeling (GJAM) was used to identify the core microbiome of wild rice that was recruited and significantly enriched under different soil conditions. The letter preceding each taxonomic name indicates the level of classification: p = phylum, c = class, o = order, f = family, g = genus, s = species

**Fig. 2:**
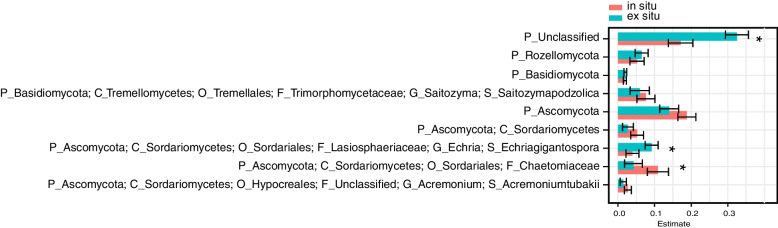
The core rhizosphere fungi of wild rice grown in situ and ex situ. Generalized joint attribute modeling (GJAM) was used to identify the core microbiome of wild rice that was recruited and significantly enriched under different soil conditions. The letter preceding each taxonomic name indicates the level of classification: p = phylum, c = class, o = order, f = family, g = genus, s = species

We used GJAM to determine the core bacteria and fungi detected in the rhizosphere that were related to genotype, excluding the influence of rhizosphere soil chemical properties. A total of 44 ASVs were obtained: 35 bacterial (Fig. 1) and 9 fungal (Fig. 2). The bacterial ASVs belonged to the phyla Actinobacteria (13 ASVs), Chloroflexi (6 ASVs), Firmicutes (1 ASV), Gemmatimonadetes (1 ASV), Nitrospirae (1 ASV), Proteobacteria (12 ASVs), and Rokubacteria (1 ASV). The relative abundance of a single bacterial ASV belonging to the class Acidimicrobiia was significantly higher (*p* < 0.05) in the rhizosphere of in situ wild rice than in the rhizosphere of ex situ wild rice (Fig. 1), while 9 ASVs belonging to the genera *Haliangium*, *Anaeromyxobacter*, *Bradyrhizobium*, *Bacillus*, and *Conexibacter*, family *Beijerinckiaceae*, and class Anaerolineae were significantly more abundant (*p* < 0.05) in the rhizosphere of ex situ wild rice. The 9 core fungal ASVs belonged to the phyla Ascomycota (5 ASVs), Basidiomycota (2 ASVs), Rozellomycota (1 ASV), and unclassified fungi (1 ASV). Four ASVs belonging to Ascomycota and one to Basidiomycota were more abundant in the rhizosphere of in situ wild rice, while four (1 unclassified, 1 Rosellomycota, 1 Basidiomycota, and 1 Ascomycota) were more abundant in the rhizosphere of ex situ wild rice (Fig. 2).

We further predicted the ecological function of the core rhizobacterial community using FAPROTAX. Five functions of the core bacteria in the rhizosphere were shared between in situ and ex situ wild rice: nitrogen fixation, manganese oxidation, aerobic chemoheterotrophy, chemoheterotrophy, and iron respiration (Table 1). The relative abundance of nitrogen fixation was highest in the rhizosphere of the STSI population of in situ wild rice and lowest in the rhizosphere of the AJS population of ex situ wild rice (*p* < 0.05). The relative abundance of aerobic chemoheterotrophy and chemoheterotrophy was highest in the rhizosphere of the STSI population of in situ wild rice, but the differences in these functions among the ex situ populations of wild rice were not significant. The relative abundance of manganese oxidation in the rhizosphere was higher for ex situ wild rice than for in situ wild rice. There was no significant difference in the relative abundance of iron respiration in the rhizosphere between in situ and ex situ wild rice.

**Table 1:** Relative abundances of the dominant predicted rhizobacterial functions in the rhizospheres of wild rice grown in situ and ex situ

Population	Nitrogen fixation (%)	Manganese oxidation (%)	Aerobic chemoheterotrophy (%)	Chemoheterotrophy (%)	Iron respiration (%)
AJSI	10.44±1.13^ab^	4.04±2.55^d^	16.60±2.59^b^	16.60±2.59^b^	27.15±6.40^a^
STSI	11.89±6.66^a^	9.83±21.16^cd^	33.95±7.75^a^	33.95±7.75^a^	4.48±2.94^a^
ZTI	3.89±6.66^bc^	11.93±15.44^cd^	17.76±19.68^ab^	17.76±19.68^ab^	31.82±42.91^a^
AJS	0.44±0.61^c^	35.87±4.61^b^	21.06±2.69^ab^	21.06±2.69^ab^	14.55±4.11^a^
STS	3.41±0.60^bc^	66.58±2.71^a^	7.55±1.23^b^	7.55±1.23^b^	11.60±1.56^a^
ZT	10.86±1.94^ab^	28.53±3.73^bc^	19.84±2.17^ab^	19.84±2.17^ab^	8.53±2.77^a^

The data in the table are the mean ± standard deviation, and different lowercase letters indicate significant (*ρ* < 0.05) differences by one-way ANOVA with Tukey’s test

Wild rice samples were collected from three in situ natural reserves, Zhangtang (ZTI), Anjiashan (AJSI), and Shuitaoshu (STSI), and three ex situ populations, Zhangtang (ZT), Anjiashan (AJS), and Shuitaoshu (STS)

### The relationship between core rhizomicrobiome function and the physicochemical properties of the soil surrounding the roots

The physicochemical properties of the soil before the wild rice was transplanted ex situ and the soil surrounding the roots of the in situ and ex situ wild rice populations are presented in Table 2. Soil pH, electrical conductivity (EC), soil organic matter (SOM), available phosphorus (AP), and available potassium (AK) differed significantly between in situ and ex situ wild rice. pH, total nitrogen (TN), SOM, and AP differed significantly among the three in situ populations. Among the three ex situ populations, TN differed significantly, whereas pH, EC, SOM, AP, and AK were not significantly different.

**Table 2:** Differences in the physical and chemical properties of the soil surrounding the roots between all populations and bulk soil before ex situ transplantation

Population	pH	EC (μS cm^−1^)	TN (g kg^−1^)	SOM (g kg^−1^)	AP (μg g^−1^)	AK (μg g^−1^)
ZTI	5.22±0.03^b^	42.62±0.58^c^	0.97±0.03^ab^	38.17±5.23^bc^	33.17±0.61^b^	61.03±11.31^a^
AJSI	5.38±0.12^a^	51.44±10.35^c^	0.77±0.01^c^	36.81±0.79^bc^	32.8±2.16^b^	52.67±1.1^ab^
STSI	4.98±0.08^c^	41.41±6.47^c^	0.9±0.05^abc^	44.61±6.26^ab^	36.84±3.62^a^	58.95±0.76^a^
BS	5.43±0.12^a^	41.84±3.86^c^	0.75±0.06^c^	42.40±4.65^ab^	13.46±1.34^c^	34.14±4.72^cd^
ZT	5.5±0.01^a^	88.7±2.16^a^	1.01±0.04^a^	46.48±5.5^a^	14.1±0.92^c^	30.87±5.49^cd^
AJS	5.44±0.07^a^	85.4±2.81^a^	0.85±0.05^bc^	26.34±2.44^d^	6.09±1.09^d^	29.22±6.74^d^
STS	5.37±0.07^ab^	72.78±0.39^b^	0.55±0.18^d^	31.88±0.8^cd^	16.98±0.47^c^	42.09±1.46^bc^

The data in the table are the mean ± standard deviation, and different lowercase letters indicate significant (*ρ* < 0.05) differences by one-way ANOVA with Tukey’s test

*EC* electrical conductivity, *TN* total nitrogen, *SOM* soil organic matter, *AP* available phosphorus, *AK* available potassium

Wild rice samples were collected from three in situ natural reserves, Zhangtang (ZTI), Anjiashan (AJSI), and Shuitaoshu (STSI), bulk soil before ex situ transplantation (BS), and three ex situ populations, Zhangtang (ZT), Anjiashan (AJS), and Shuitaoshu (STS)

**Fig. 3:**
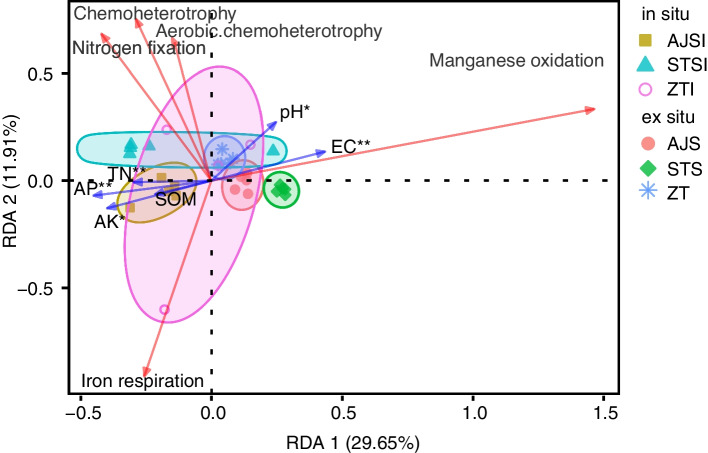
Redundancy analysis (RDA) of the functions of the core bacterial community in the rhizosphere of wild rice as explained by the physicochemical properties of the soil surrounding the roots. EC = electric conductivity; TN = total nitrogen; SOM = soil organic matter; AP = available phosphorus; AK = available potassium

Redundancy analysis (RDA) of the functions of the core bacterial community in the rhizosphere of wild rice revealed that the first canonical axis explained 29.65% of the variation in soil physicochemical properties, while the second canonical axis explained 11.91% of the total variation (Fig. 3). EC, TN, and AP had extremely significant (*p* < 0.01) roles in shaping the function of the core bacterial community in the rhizosphere of wild rice. AK and pH were significantly correlated (*ρ* < 0.05) with the function of the wild rice rhizosphere core bacterial community. Manganese oxidation was positively correlated with EC and negatively correlated with TN, AP, and AK. In addition, pH was positively correlated with aerobic chemoheterotrophy.

### Residual correlations between the core rhizomicrobiome and the physicochemical properties of the soil surrounding the roots

**Fig. 4:**
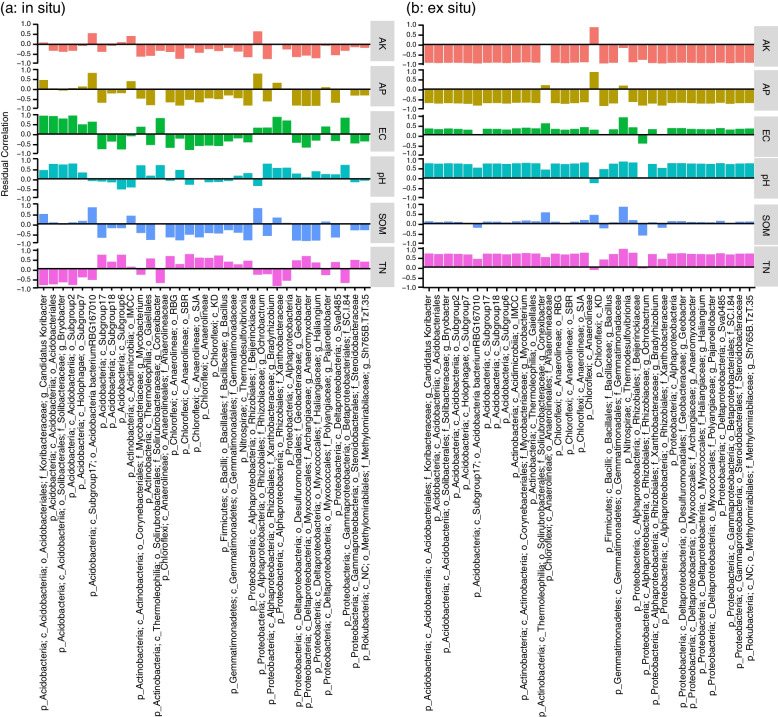
The relationship between the physicochemical properties of the soil surrounding the roots and the core rhizosphere bacteria of wild rice grown in situ (**a**) and ex situ (**b**). Generalized joint attribute modeling (GJAM) was used to identify correlations between soil properties and the core rhizosphere bacteria of wild rice. The letter preceding each taxonomic name indicates the level of classification: p = phylum, c = class, o = order, f = family, g = genus, s = species. The order of the taxonomic names is the same in **a** and **b**

Analysis of the residual correlations revealed that AK was negatively correlated with *Bryobacter*, Acidobacteria subgroup 2, Holophagae subgroup 7, *Mycobacterium*, Gaiellales, *Anaerolineaceae*, *Bacillus*, *Gemmatimonadaceae*, *Ochrobacterium*, and *Geobacter* and positively correlated with *Candidatus Koribacter*, Acidobacteria, Acidobacteria subgroup 6, Acidimicrobiia, and *Beijerinckiaceae* in the rhizosphere of wild rice grown in situ (Fig. 4). AP was negatively correlated with Acidobacteria subgroup 17, Acidobacteria subgroup 18, Acidimicrobiia, *Mycobacterium*, Gaiellales, *Anaerolineaceae*, Anaerolineae, *Bacillus*, *Gemmatimonadaceae*, *Ochrobacterium*, *Geobacter*, and *Haliangium* and positively correlated with *Candidatus Koribacter*, Acidobacteria subgroup 7, Acidobacteria subgroup 6, Acidimicrobiia, *Beijerinckiaceae*, *Bradyrhizobium*, and *Pajaroellobacter* in the rhizosphere of wild rice grown in situ.

Twenty core bacterial ASVs were negatively correlated with EC in the rhizosphere of wild rice grown in situ; by contrast, in the rhizosphere of wild rice grown ex situ, EC was negatively correlated with only one ASV (*Ochrobacterium*) and was positively correlated with the remaining 19 ASVs. Only Anaerolineae was negatively correlated with pH in the rhizosphere of in situ wild rice, while the other core bacteria were positively correlated with pH in the rhizosphere of ex situ wild rice. Interestingly, the residual correlation index between the core bacteria and the SOM in the soil surrounding the roots was higher for in situ wild rice than for ex situ wild rice. Fourteen core bacterial ASVs were negatively correlated with TN in the rhizosphere of wild rice grown in situ. By contrast, in the rhizosphere of ex situ wild rice, only one bacterial core ASV was negatively correlated with TN, and the rest were positively correlated with TN.

**Fig. 5:**
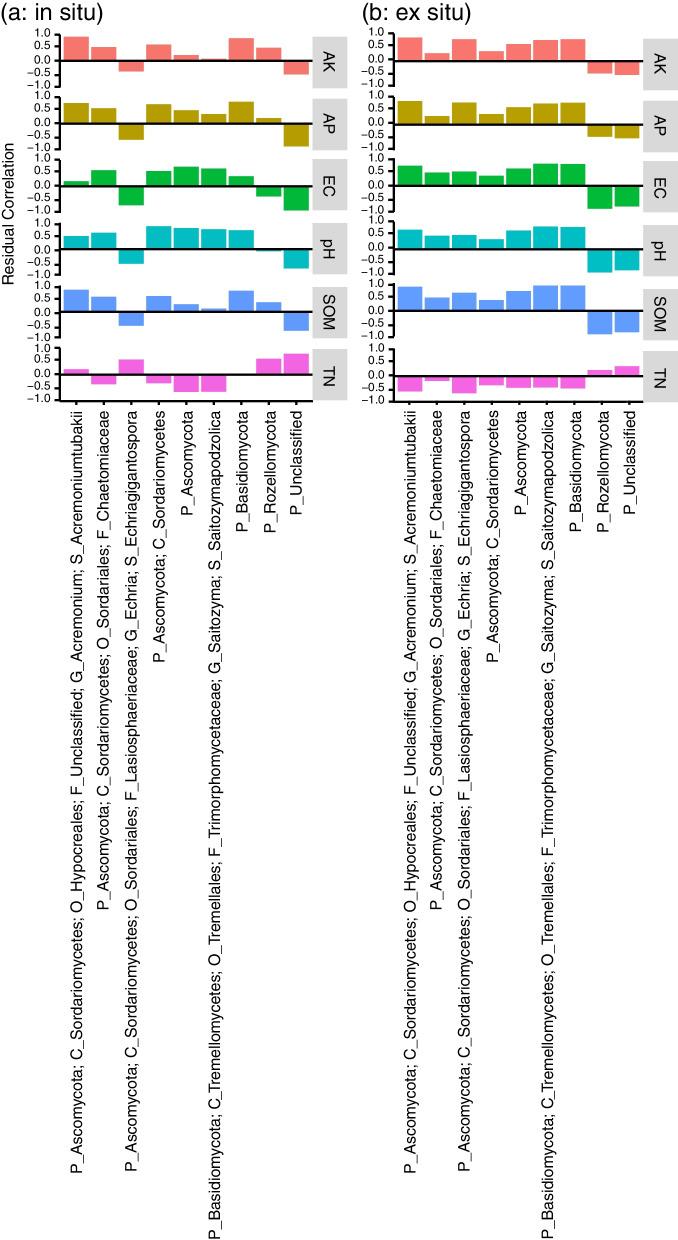
The relationship between the physicochemical properties of the soil surrounding the roots and the core rhizosphere fungi of wild rice grown in situ (**a**) and ex situ (**b**). Generalized joint attribute modeling (GJAM) was used to identify correlations between soil properties and the core rhizosphere fungi of wild rice. The letter preceding each taxonomic name indicates the level of classification: p = phylum, c = class, o = order, f = family, g = genus, s = species. The order of the taxonomic names is the same in **a** and **b**

The residual correlations between the core fungi and the physicochemical properties of the soil surrounding the roots were similar between in situ and ex situ wild rice (Fig. 5). Notably, *Echriagigantospora* was negatively correlated with AK, AP, EC, pH, and SOM and positively correlated with TN in the rhizosphere of in situ wild rice, whereas opposing correlations were observed in the rhizosphere of ex situ wild rice.

## Discussion

Ecologists have long been fascinated by the core microbiome in the soil and rhizosphere, which plays a key role in crop growth and health. Studies have sought to identify and understand the core microbiome in the rhizosphere [23, 31] using various methods, including Venn diagrams, linear discriminant analysis effect size (LEfSe) based on the linear discriminant analysis (LDA) score, co-occurrence based on Spearman and Pearson correlation analyses, and combinations of two or three methods [22, 23, 32]. Each of these methods has specific drawbacks; for example, Venn diagrams only identify ASVs that are detected in all treatments and do not consider relative abundances [22]. Co-occurrence analysis investigates the rhizosphere microbial community under a compositional constraint (the limitation imposed by sequencing) using Spearman and Pearson correlation analyses, resulting in a lack of independence of the correlation analysis [33]. In their recent review, Leite and Kuramae [25] provided a good overview of model-based approaches, including GJAM, that can explicitly account for key statistical properties of data. GJAM considers the observed amplicon sequences as censored versions of the true abundance, thus accounting for the compositional constraint [34]. In the present study, we used GJAM to identify the microbes consistently selected by the wild rice genotype regardless of soil conditions (in situ vs ex situ). We call these microbes the core selection of wild rice recruited under different soil conditions. As selection criteria, we considered regression coefficients that were (i) significantly different from zero and (ii) positive regardless of soil conditions. In a recent study, Rotoni et al. [35] used the same method to reveal the “core selection”—a synonym of “core microbiome”—of the rhizosphere microbiome that is recruited regardless of host genetic variability. Together, these selection criteria provided a statistical background for the reliable identification of microbes consistently selected by the different populations of wild rice.

Nine core bacterial ASVs were more abundant in the rhizosphere of ex situ wild rice; these ASVs belonged to the genera *Haliangium*, *Anaeromyxobacter*, *Bradyrhizobium*, *Bacillus*, and *Conexibacter*, the family *Beijerinckiaceae*, and the class Anaerolineae. By contrast, only one ASV belonging to the class *Acidimicrobiia* was more abundant in the rhizosphere of in situ wild rice. One possible explanation for this result is that the residual correlations of these core ASVs with soil properties differed between in situ and ex situ wild rice. For instance, Acidimicrobiia were positively correlated with pH and EC and negatively correlated with AP and AK in the rhizosphere of in situ wild rice, but after long-term transplantation ex situ, these relationships shifted to negative and positive correlations, respectively. Numerous studies have demonstrated that soil pH, nutrients, and organic matter are the most important soil-related factors determining the composition and structure of the rhizosphere microbiome [10, 12, 36, 37], and our results show that this extends to the soil surrounding the roots of wild rice populations. Acidimicrobiia can oxidize Fe^2+^ into Fe^3+^, and colloidal Fe^3+^ becomes the protective layer of iron phosphate [38]. Interestingly, *Bacillus* help plant hosts develop tolerance to pathogens and pests [39] and reduce oxidized Mn^4+^ to Mn^2+^ [40], consistent with the greater potential function of manganese oxidation in the rhizosphere of ex situ wild rice. *Conexibacter* are saccharolytic; i.e., they receive carbon and energy from carbohydrate hydrolysis [41]. Additionally, the genera *Anaeromyxobacter*, *Bradyrhizobium*, and *Conexibacter*, the family *Beijerinckiaceae*, and the class Anaerolineae represent free-living N fixers [42]. Nitrogen use efficiency (NUE) in rice paddies is only 30–45% due to losses from ammonia (NH_3_) volatilization, surface runoff, nitrification-denitrification, and leaching [43]. N_2_O emissions from paddy fields in China have been estimated to account for 20% of global N_2_O emissions [44]. Future studies may explore the use of core bacteria of wild rice to promote plant growth and reduce greenhouse gas (GHG) emissions in combination with soil factors (nutrient input dosages) in domesticated rice cultivation.

Domestication has been shown to alter the diversity of microbes in the rhizosphere of crops such as corn [45], tomato [46], wheat [47], and rice [48, 49]. Chang et al. [50] compared the bacterial communities of wild and domesticated rice genotypes and found that *Frankiaceae* was enriched in the rhizosphere of cultivated *Oryza sativa* but absent from the rhizosphere of wild *Oryza rufipogon*. This result highlights that unique microbes that are not recruited by wild relatives are enriched in the rhizosphere of domesticated cultivars. Similarly, Chanco et al. [51] showed that wild tomatoes grown in native soils harbor unique beneficial root microbiota at higher abundances than modern tomato cultivars.

Prediction of the ecological functions of the core rhizosphere bacterial community using FAPROTAX identified nitrogen fixation, manganese oxidation, aerobic chemoheterotrophy, chemoheterotrophy, and iron respiration. Manganese oxidation, which plays an important role in the manganese cycle in the rhizosphere ecosystem [40, 44], was enriched in the rhizosphere of ex situ wild rice compared with the rhizosphere of in situ wild rice. RDA indicated that the manganese cycle was positively correlated with EC and negatively correlated with TN, AP, and AK. Aerobic chemoheterotrophy and chemoheterotrophy, which involve organic carbon metabolism and are closely related to the circulation of organic matter and flow of energy in the system [52, 53], were significantly enriched in the rhizosphere of wild rice grown in STSI compared with the other in situ wild rice populations, whereas these functions did not differ significantly among the ex situ populations. Aerobic chemoheterotrophy was also positively correlated with rhizosphere soil pH. The relationship between carbon and iron is affected by microbial iron respiration according to the following reaction: 2Fe_2_O_3_∙ nH_2_O + CH_2_O + 7H^+^ → 4Fe^2+^ + HCO^3−^ + (2n + 4)H_2_O + chemical energy [54]. Generally, Fe^3+^ is absorbed by plant roots, and positron-emitting tracer imaging has shown that rice takes up both Fe^3+^ and Fe^2+^ [55]. In addition, iron respiration coincides with strong suppression of methanogenesis [56]. The microbial iron respiration function of the wild rice core rhizobacterial community might suppress methane production, which would corroborate our earlier finding that methane metabolism is higher in the rhizosphere of domesticated rice than in the rhizosphere of wild rice [57]. Interestingly, nitrogen fixation was identified as a function of the core rhizobacterial community in both in situ and ex situ wild rice, suggesting that wild rice growth relies on the rhizomicrobiome for nitrogen nutrient uptake. In summary, the potential functions of the core microbiome independent of growing site (in situ or ex situ) are related to resource acquisition for wild rice growth. However, the predicted functions were based on partial 16S rRNA gene sequences; the functions of the microbiota in the rhizosphere could be more accurately determined using target approaches such as quantitative real-time PCR of functional genes of interest or general functional profiling via shotgun metagenomic sequencing.

As a first step in developing novel systems to use the core rhizosphere microbiome to improve rice growth, we recommend performing a detailed analysis of the chemical microenvironment surrounding the root to systematically study the impact of soil properties on the plant-microbe association. Insights gained from such studies of wild rice plants can be used to strengthen the role of the core rhizomicrobiome of cultivated rice to improve the sustainability of rice production. In the present study, GJAM showed that soil properties play a key role in the abundances of the core microbiome. For instance, *Bradyrhizobium* were positively correlated with AP in the rhizosphere of in situ wild rice, but after long-term transplantation ex situ, this relationship shifted to a negative correlation. Nitrogen fixation by bacteria such as *Bradyrhizobium* is typically hampered by low phosphorus availability in N-fixing grain legumes [58]. These opposing correlations might reflect the significantly higher abundance of *Bradyrhizobium* in the rhizosphere of ex situ wild rice compared with in situ wild rice, suggesting a threshold value of abundance that controls the correlation with AP. When the abundance of *Bradyrhizobium* is low, increasing the concentration of available phosphorus may increase the recruitment of N fixers; by contrast, when the abundance of *Bradyrhizobium* is high enough, increasing available phosphorus may inhibit the growth and recruitment of N fixers. Taken together, the results of RDA, GJAM, and soil property analysis indicate that small differences in soil chemical factors underlie changes in the relative abundances of the core microbiome and function. However, this conclusion awaits verification by experiments. Future work will explore the threshold values of the properties of the soil surrounding the roots for controlling the correlations of such properties with the core microbiome, especially relationships with the soil availability of nutrients.

## Conclusion

The importance of the interaction between crop domestication and the microbiome is increasingly apparent, and food production research is beginning to clarify the mechanisms by which wild crops harbor unique microbiomes so that these microbiomes can be applied to modern cultivated crops [59, 60]. The current work presents the first snapshot of the core rhizosphere microbiome of wild rice under both in situ and ex situ long-term field conditions. The findings indicate that the rhizosphere microbiome holds great potential for domestication. Most previous studies have analyzed the microbiome attached to seeds/seedlings of crops in pots under greenhouse conditions, whereas this study was performed under field conditions, which better supports the applicability of the findings [45, 61, 62]. Furthermore, the comparison of in situ and ex situ populations provides more solid proof for the positive selection of the microbiome by wild rice rather than by the soil, especially given the 40-year history of ex situ cultivation. The generalized joint attribute model (GJAM), a powerful identification tool, was applied here to identify and quantify the core microbiome [35]. The predicted ecological functions of the core rhizosphere bacteria were nitrogen fixation, manganese oxidation, aerobic chemoheterotrophy, chemoheterotrophy, and iron respiration, suggesting that the core microbiome in the rhizosphere of wild rice has the potential to improve the acquisition of nitrogen, manganese, and organic carbon resources for rice growth. The EC, TN, AP, pH, and AK of the soil surrounding the roots were found to have significant roles in shaping the relative abundances of the functions of the core bacterial community in the wild rice rhizosphere. Overall, the first core microbiome of wild rice presented here paves the way for a deeper investigation of the interaction between rice domestication and the microbiome and for the application of the core microbes of wild rice populations to domesticated rice as potential bioinoculants to increase resource acquisition (nitrogen, manganese, iron, carbon) for sustainable rice production and global food security.

## Material and methods

### Sampling method

Dongxiang wild rice (*Oryza rufipogon*) populations were sampled from three in situ natural reserves in Dongxiang City, Jiangxi Province, China, i.e., Zhangtang (ZTI), Anjiashan (AJSI), and Shuitaoshu (STSI), and three ex situ artificial protection nurseries in Nanchang City, Jiangxi Province, China, i.e., Zhangtang (ZT), Anjiashan (AJS), and Shuitaoshu (STS) (Fig. S3). The in situ and ex situ populations have all been protected for more than 40 years and the soil was classified as a Latosol according to the United States Department of Agriculture (USDA) soil classification system. The study area, Jiangxi Province, China, is characterized by a humid subtropical monsoon climate. The mean annual precipitation (MAP) is 1593.6 mm, and the mean annual temperature (MAT) is 17.2 °C [28]. For each of the wild rice populations, 5 plots (1 m^2^) were selected for sampling the wild rice rhizosphere. Five plants (“S” distribution) were selected and mixed as a biological replicate, and five replicates were obtained from each plot. The rhizosphere soil and the loose soil surrounding the roots were collected for use in DNA sequencing and soil physicochemical property evaluation, respectively. To collect the rhizosphere soil, the root was immersed in a tube containing 5 ml of sterile water to collect the 1-mm layer of soil surrounding the root. The tube was centrifuged at a relative centrifugal force of 10,000×*g* for 30 s, and after removal of the supernatant, the rhizosphere soil sample was stored at −80 °C. In addition, the loose soil surrounding the root was collected, air dried, and passed through a 2-mm sieve prior to physical and chemical analyses.

### DNA extraction and amplicon sequencing

DNA was extracted from 0.5 g of rhizosphere soil according to the instructions of the Fast DNA SPIN kit (MP Biomedicals, Eschwege, Germany). The bacterial V3–V4 hypervariable region and fungal ITS1 were amplified using the primers 338F (5′-ACTCCTACGGGAGGCAGCA-3′) and 806R (5′-GGACTACHVGGGTWTCTAAT-3′) [63] and ITF5F (5′-GGAAGTAAAAGTCGTAACAAGG-3′) and ITS1R (5′-GCTGCGTTCTTCATCGATGC-3′) [64], respectively. The PCR program was as follows: initial denaturation at 98 °C for 2 min; 25 cycles of denaturation at 98 °C for 15 s, annealing at 55 °C for 30 s and extension at 72 °C for 30 s; and a final extension step at 72 °C for 10 min. The PCR amplicons were purified and quantified using Agencourt AMPure Beads (Beckman Coulter, Indianapolis, IN, USA) and the PicoGreen dsDNA Assay Kit (Invitrogen, Carlsbad, CA, USA), respectively, and used in 250-bp paired-end sequencing on the Illumina HiSeq 2500 PE250 platform (Biomarker Technologies Co. Ltd., Beijing, China). The raw reads were filtered using Trimmomatic v 0.33 software, and Cutadapt 1.9.1 software was used to identify and remove primer sequences to obtain clean reads [65]. Paired-end reads were assembled using Usearch v10. To obtain representative sequences, the sequences were denoised using the Dada2 plugin in QIIME2 software as described by Callahan et al. [66]. ASV taxonomic classification was conducted by BLAST searching the representative sequence set against the SILVA database for bacteria (version 132, https://www.arb-silva.de/aligner/) and the UNITE database for fungi (version 5.0, https://unite.ut.ee/analysis.php/) [67, 68]. Potential functions in the rhizosphere bacterial community were determined using the FAPROTAX database [69, 70].

### Analysis of rhizosphere soil physical and chemical properties

Soil pH and electrical conductivity (EC) were measured in a suspension of soil in water at a ratio of 1:2.5 (w/v). Soil total nitrogen (TN), soil organic matter (SOM), available phosphorus (AP), and available potassium (AK) were measured by the Kjeldahl method, potassium dichromate volumetric method, NaHCO_3_ extraction method and ammonium acetate extraction method, respectively, as described by Chang et al. [50].

### Statistical analysis

Principal coordinate analysis (PCoA) was used to visualize differences in the wild rice rhizosphere bacterial and fungal communities between the in situ and ex situ populations based on the Bray–Curtis dissimilarity matrix using the “vegan” package in R (v3.6.2). The statistical significance of the clustering patterns in the ordination plots was subsequently evaluated by PERMANOVA. To evaluate the number of shared and significantly enriched ASVs across the in situ and ex situ wild rice rhizospheres, we used the “gjam” package in R (v4.1.2). Generalized joint attribute modeling (GJAM) permitted the identification of the core microbiome of wild rice recruited under different soil conditions and the inference and interpretation of the relationships between different groups of variables from the residual correlations (e.g., soil properties and the rhizosphere microbiome) on the observation scale while avoiding distorted correlations [25, 26]. Regression coefficients defining the relative abundances of microbiomes were extracted for all populations. The core rhizomicrobiome comprised microbes with regression coefficients that were positive and significantly different from zero for the different populations and soil conditions. The relationships between the functions of this core microbiome and soil properties in the rhizosphere were examined using redundancy analysis (RDA) with the “vegan” package in R (v3.6.2).

## Supplementary Information

**Additional file 1: Table S1**. Statistics for the quality assessment of the sequencing data.

**Additional file 2: Table S2**. Details of the sampling sites.

**Additional file 3: Figure S1**. The α-diversity of the (A, B) bacterial and (C, D) fungal communities in the rhizosphere of wild rice populations of the in situ natural reserves (ZTI, Zhangtang; AJSI, Anjiashan; STSI, Shuitaoshu) and ex situ (ZT, Zhangtang; AJS, Anjiashan; STS, Shuitaoshu).

**Additional file 4: Figure S2**. The principal coordinate analysis (PCoA) of the (A) bacterial and (B) fungal communities in the rhizosphere of wild rice populations of the in situ natural reserves (ZTI, Zhangtang; AJSI, Anjiashan; STSI, Shuitaoshu) and ex situ (ZT, Zhangtang; AJS, Anjiashan; STS, Shuitaoshu).

**Additional file 5: Figure S3**. The wild rice sites. The three in situ natural reserve sites (A) AJSI, (B) STSI and (C) ZTI and three ex situ artificial protection nurseries (D) AJS, (E) STS and (F) ZT. (In Figure C, professor Dazhou Chen who is one of the initiators for Dongxiang wild rice conservation is observing the growth status of wild rice).

## Data Availability

The sequences were deposited in the National Center for Biotechnology Information (NCBI; https://www.ncbi.nlm.nih.gov/) under accession number PRJNA830921.
