# Supplementary material for: Nitrogen, manganese, iron, and carbon resource acquisition are potential functions of the wild rice Oryza rufipogon core rhizomicrobiome
Source: Microbiome. 2022 Nov 22;10:196. doi: 10.1186/s40168-022-01360-6 (PMC9682824; doi:10.1186/s40168-022-01360-6)
Supplement: Supplementary file 2 — Additional file 1: Table S1. Statistics for the quality assessment of the sequencing data. [file 40168_2022_1360_MOESM1_ESM.docx]

Table S1 Statistics for the quality assessment of the sequencing data.

| Kingdom | Population | Raw Reads | Clean Reads | Denoised Reads | Merged Reads | Non-chimeric Reads |
| --- | --- | --- | --- | --- | --- | --- |
| Bacteria | AJSI | 159,504 | 128,994 | 111,602 | 51,267 | 43,600 |
|  | STSI | 177,061 | 141,245 | 131,245 | 88,284 | 81,357 |
|  | ZTI | 179,358 | 146,724 | 134,794 | 90,474 | 83,122 |
|  | AJS | 170,958 | 134,882 | 117,503 | 56,114 | 49,137 |
|  | STS | 162,704 | 128,889 | 115,287 | 69,670 | 62,926 |
|  | ZT | 178,052 | 139,995 | 125,969 | 61,284 | 52,638 |
| Fungi | AJSI | 289,737 | 133,519 | 130,141 | 124,585 | 122,913 |
|  | STSI | 57,396 | 36,174 | 35,300 | 33,604 | 33,164 |
|  | ZTI | 46,800 | 25,259 | 24,593 | 23,340 | 23,020 |
|  | AJS | 249,833 | 123,813 | 121,056 | 117,362 | 116,417 |
|  | STS | 50,728 | 25,942 | 25,386 | 24,579 | 24,265 |
|  | ZT | 334,018 | 160,182 | 156,511 | 147,821 | 143,622 |

Raw Reads: the number of original sequences; Clean Reads: the number of optimized reads filtered from the raw reads; Denoised Reads: the number of reads after denoising of clean reads; Merged Reads: the number of sequences obtained after splicing of the denoised reads according to overlap; Non-chimeric Reads: the number of sequences after the final removal of chimeras.

Wild rice samples were collected from three in situ natural reserves, Zhangtang (ZTI), Anjiashan (AJSI), and Shuitaoshu (STSI), and three ex situ populations, Zhangtang (ZT), Anjiashan (AJS), and Shuitaoshu (STS).
