# Supplementary material for: Nitrogen, manganese, iron, and carbon resource acquisition are potential functions of the wild rice Oryza rufipogon core rhizomicrobiome
Source: Microbiome. 2022 Nov 22;10:196. doi: 10.1186/s40168-022-01360-6 (PMC9682824; doi:10.1186/s40168-022-01360-6)
Supplement: Supplementary file 3 — Additional file 2: Table S2. Details of the sampling sites. [file 40168_2022_1360_MOESM2_ESM.docx]

Table S2 Details of the sampling sites.

| Sampling site | Longitude and latitude | Altitude (m) |
| --- | --- | --- |
| AJSI | N28°6′57″ E116°32′53″ | 68.59 |
| STSI | N28°6′10″  E116°31′12″ | 50.33 |
| ZTI | N28°5′42″  E116°32′24″ | 501.01 |
| Ex situ | N28°33′31″  E115°56′21″ | 40.16 |

Wild rice samples were collected from three in situ natural reserves, Zhangtang (ZTI), Anjiashan (AJSI), and Shuitaoshu (STSI) located in different positions, and three ex situ populations were located in the same field position.
