# Supplementary figures and images for: Nitrogen, manganese, iron, and carbon resource acquisition are potential functions of the wild rice Oryza rufipogon core rhizomicrobiome
Source: Microbiome. 2022 Nov 22;10:196. doi: 10.1186/s40168-022-01360-6 (PMC9682824; doi:10.1186/s40168-022-01360-6)

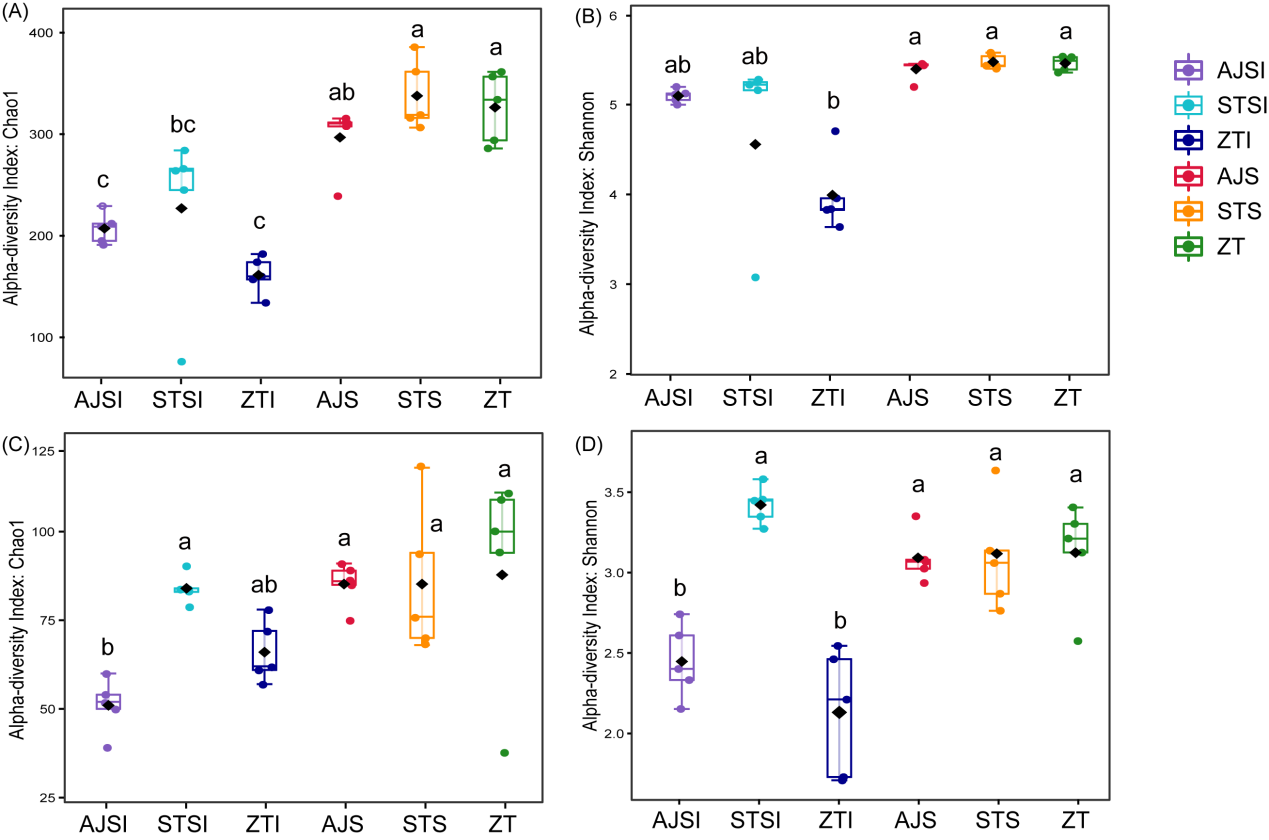

Supplement: Supplementary file 4 — Additional file 3: Figure S1. The α-diversity of the (A, B) bacterial and (C, D) fungal communities in the rhizosphere of wild rice populations of the in situ natural reserves (ZTI, Zhangtang; AJSI, Anjiashan; STSI, Shuitaoshu) and ex situ (ZT, Zhangtang; AJS, Anjiashan; STS, Shuitaoshu). [file 40168_2022_1360_MOESM3_ESM.docx]

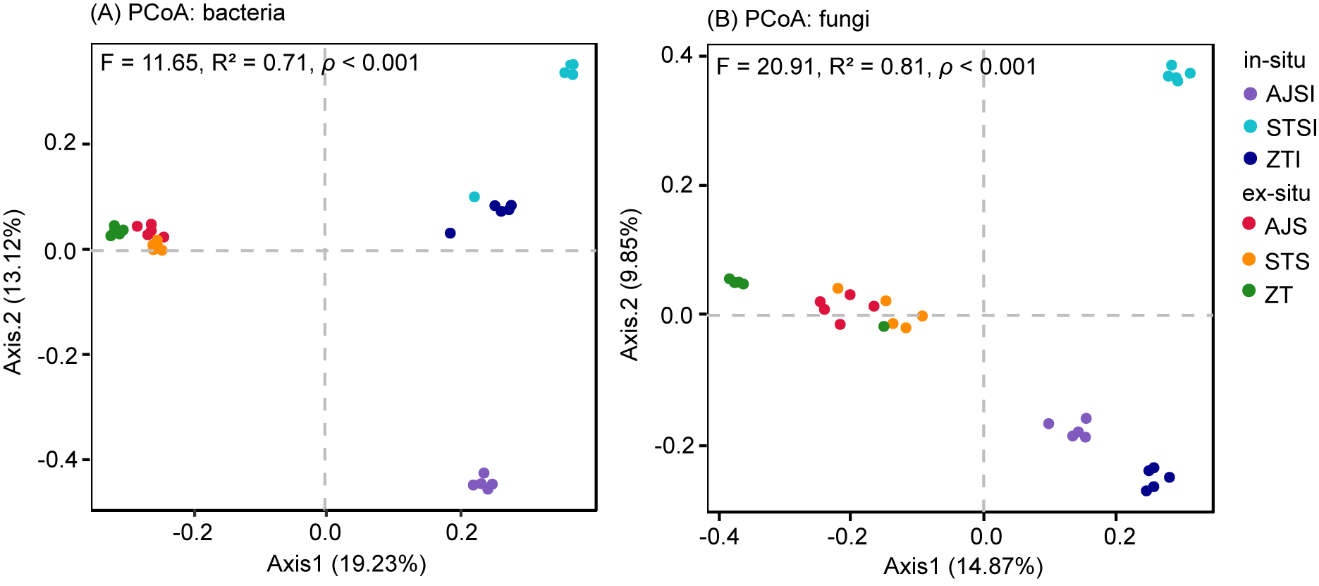

Supplement: Supplementary file 5 — Additional file 4: Figure S2. The principal coordinate analysis (PCoA) of the (A) bacterial and (B) fungal communities in the rhizosphere of wild rice populations of the in situ natural reserves (ZTI, Zhangtang; AJSI, Anjiashan; STSI, Shuitaoshu) and ex situ (ZT, Zhangtang; AJS, Anjiashan; STS, Shuitaoshu). [file 40168_2022_1360_MOESM4_ESM.docx]

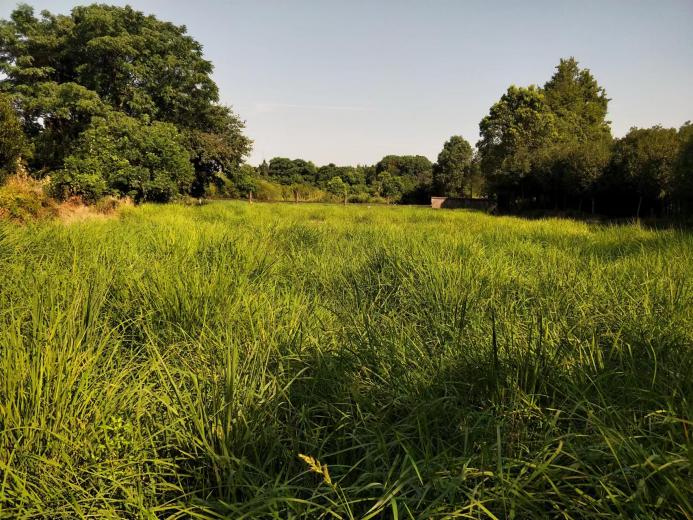


(A)


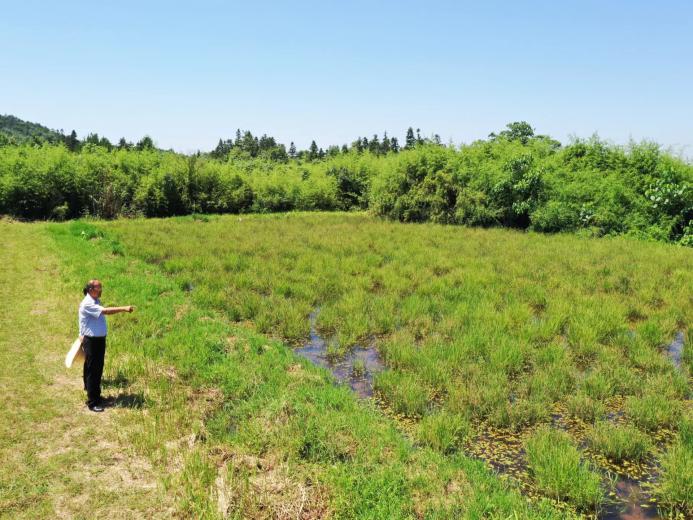


(C)


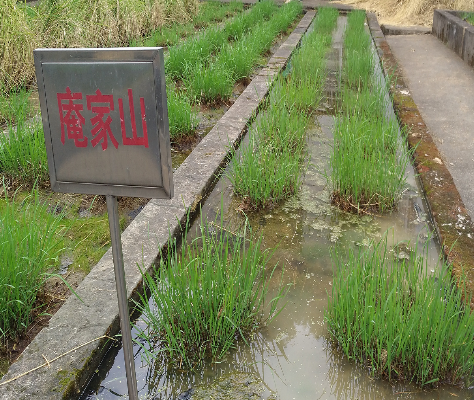


(D)


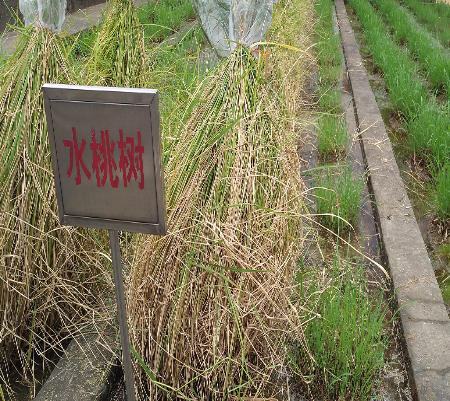


(E)


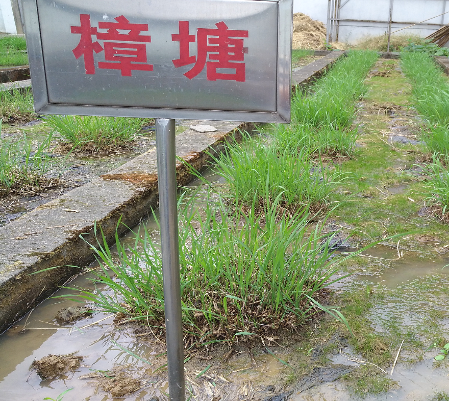


(F)

Supplement: Supplementary file 6 — Additional file 5: Figure S3. The wild rice sites. The three in situ natural reserve sites (A) AJSI, (B) STSI and (C) ZTI and three ex situ artificial protection nurseries (D) AJS, (E) STS and (F) ZT. (In Figure C, professor Dazhou Chen who is one of the initiators for Dongxiang wild rice conservation is observing the growth status of wild rice). [file 40168_2022_1360_MOESM5_ESM.docx]
